# Supplementary material for: Enhanced Intestinal TGF-β/SMAD-Dependent Signaling in Simian Immunodeficiency Virus Infected Rhesus Macaques
Source: Cells. 2021 Apr 4;10(4):806. doi: 10.3390/cells10040806 (PMC8066988; doi:10.3390/cells10040806)
Supplement: Supplementary file 1 [file cells-10-00806-s001.zip › Supplemental Figures and Table/Supplemental Table S1.pdf]

**Supplemental Table S1. List of primary antibodies used for identifying mucosal mononuclear and epithelial cells in rhesus macaques by flow cytometry and immunohistochemistry staining**

| Antibody | Isotype | Clone | Assay | Working Dilution/ amount | Source |
|---|---|---|---|---|---|
| Anti-cow Cytokeratin, Wide Spectrum Screening | Rabbit polyclonal IgG | - | IF | 1:500 | Dako |
| Anti-human CD3 | Mouse IgG1 | SP34.2 | FC | 5μl | BD Biosciences |
| Anti-human CD3 | Rabbit polyclonal | - | IF | 1:100 | Biocare Medical |
| Anti-human CD11c | Mouse IgG1 | 3.9 | IF | 1:50 | Biolegend |
| Anti-human CD4 | Mouse IgG1 | L200 | FC | 3 μl | BD Biosciences |
| Anti-human CD8 | Mouse IgG2a | 3B5 | FC | 3 μl | Invitrogen |
| Anti-human CD14 | Mouse IgG2a | M5E2 | FC | 5 μl | Biolegend |
| Anti-human CD20 | Mouse IgG2b | 2H7 | FC | 2 μl | Biolegend |
| Anti-NHP CD45 | Mouse IgG1 | D058-1283 | FC | 5 μl | BD Biosciences |
| Anti-human CD68 | Mouse IgG1 | KP1 | FC, IF | 5 μl, 1:50 | Santa Cruz Biotechnology |
| Anti-human CD79a | Mouse IgG1 | HM47 | IF | 1:100 | BD Biosciences |
| Anti-human CD159a (NKG2A) | Mouse IgG2b | Z199 | FC | 5 μl | Beckman Coulter |
| Anti-human CD163 | Mouse IgG1 | GHI/61 | FC | 5 μl | Biolegend |
| Anti-human IL-10 | Rat IgG1 | JES3-9D7 | FC | 5 μl | Biolegend |
| Anti-human Smad7 | Mouse IgG2a | Z8-B | IF | 1:50 | Santa Cruz Biotechnology |
| Anti-human TGF-β1 | Mouse IgG1 | TB21 | IHC | 1:100 | Santa Cruz Biotechnology |
| Anti-human TGF--β1 | Mouse IgG1 | 9016 | FC | 10 μl | R&D Systems |
| Anti-human TGF-βRII | Goat IgG | - | FC, IF | 10 μl, 1:100 | R&D Systems |
| Anti-human macrophage | Mouse IgM | HAM56 | IF | 1:50 | Enzo |
| Anti-human myeloid/histiocyte antigen | Mouse IgG1 | MAC387 | IF | 1:100 | Dako |
| pSMAD2/3 | Rabbit polyclonal | - | IF | 1:50 | Santa Cruz Biotechnology |
| Mouse IgG1 kappa isotype control | Mouse IgG1 | | IHC, IF | 1:100 | Invitrogen |
| Mouse IgG2a | Mouse IgG2a | | IF | 1:50 | Invitrogen |
| Normal mouse IgM | Mouse IgM | | IF | 1:50 | Santa Cruz Biotechnology |
| Normal Goat IgG | Goat IgG | | IF | 1:100 | R&D Systems |
| Normal Rabbit IgG control | Rabbit IgG | | IF | 1:100 | R&D Systems |

**Note:** Before all staining, the dilution of the respective antibodies was determined after serial dilution experiment.

FC, IF and IHC denote flow cytometry, immunofluorescence and immunohistochemistry, respectively.
