# Supplementary material for: Enhanced Intestinal TGF-β/SMAD-Dependent Signaling in Simian Immunodeficiency Virus Infected Rhesus Macaques
Source: Cells. 2021 Apr 4;10(4):806. doi: 10.3390/cells10040806 (PMC8066988; doi:10.3390/cells10040806)

Supplemental Figures:

Supplemental Figure S1. Intracellular cytokine flow cytometry for TGF-β responses from a representative SIV infected RhM. Cells were gated first on singlets, live cells and then gated based as CD3+, CD20+ and CD3–CD20– cells based on their surface CD3 and CD20 expression. Cell subsets were further gated as TGF-β+ and TGF-β- cells based on the production of TGF-β as shown in the box. The frequency in each box and in quadrant represents the frequency of that subpopulation. The bottom contour plot shows the overlay distribution of TGF-β+ cells gated from CD3–CD20– cell population. Interestingly, the majority of the cells displayed high forward and side scatter, suggesting that TGF-β+ cells are bigger in size and/or complexity or granularity like macrophages, NKs and/or dendritic cells.

Supplemental Figure S2. Mean values of the respective population positive of TGF-β+ from CD3–CD20–cells in SIV acutely infected macaques (21 dpi, n=3). Cells were gated first on singlets, live cells and then gated based as CD3+, CD20+ and CD3–CD20– cells based on their surface CD3 and CD20 expression. The TGF-β+ cells from CD3–CD20– cells were further stained for different markers (NKG2A, CD163, CD14, CD45, CD68, CD8 and IL-10) and analyzed. Note, the majority of the TGF-β+ cells from CD3–CD20– cells were CD68 positive (mean ± the standard errors, 96.3 ± 1.5).

Supplemental Figure S3. Representative expression of TGF-βRII in SIV-uninfected (FK25), control as well as acute (CF65, 21 dpi), and chronic (EB09, 250 dpi) SIV infected RhMs by multi-label confocal microscopy. The labels in each image are indicated on the Y-axis. Epithelial cells (cytokeratin+) cells were positive for TGF-βRII as represented by yellow arrows. Of note, we did not see any upregulation of TGF-βRII expression in epithelial cells among SIV-infected and uninfected control RhMs.

Supplemental Figure S4. Relative fold changes (mean ± the standard errors) of TGF-β, SMAD3, and SMAD7 gene expression are shown in jejunum tissues from female RhMs (n=3). All samples were normalized against 18S rRNA expression, and fold changes are relative to mean expression levels in male RhMs (n=3). The values above the bar represent mean flod change value for that specific gene.

Supplemental Figure S1

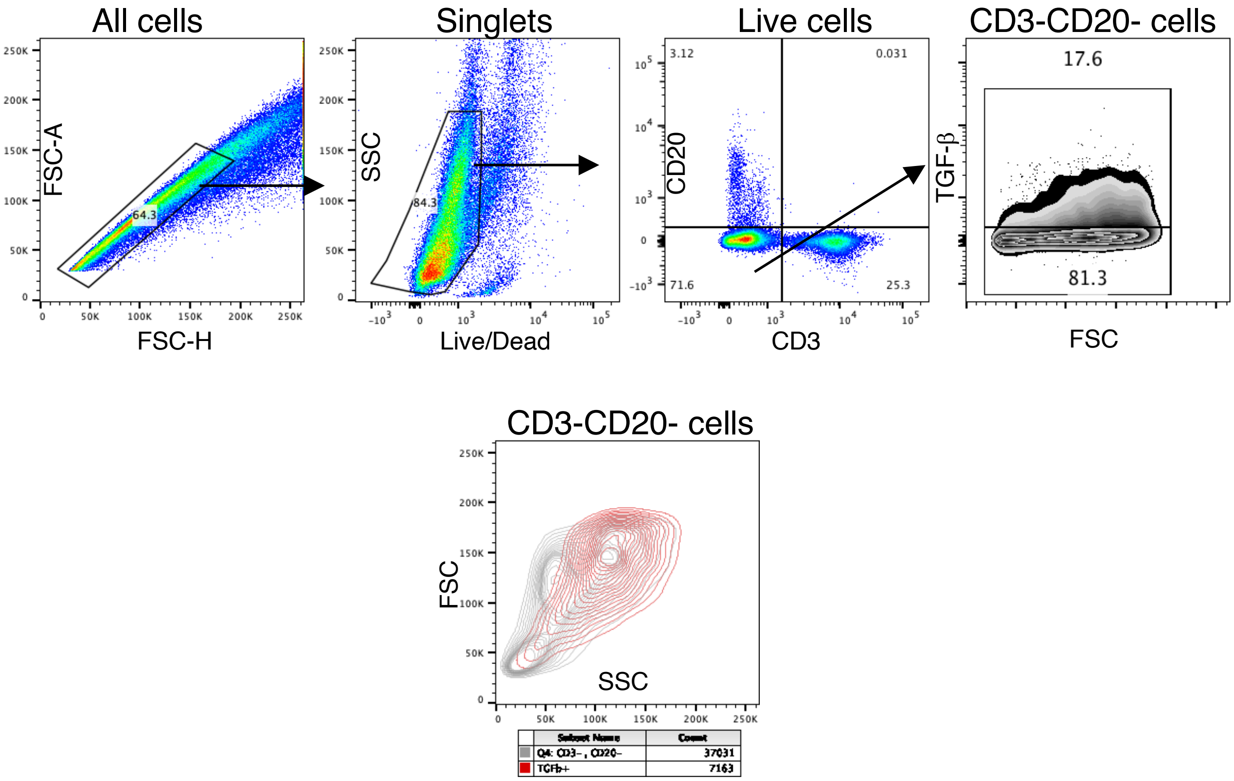

Supplemental Figure S2

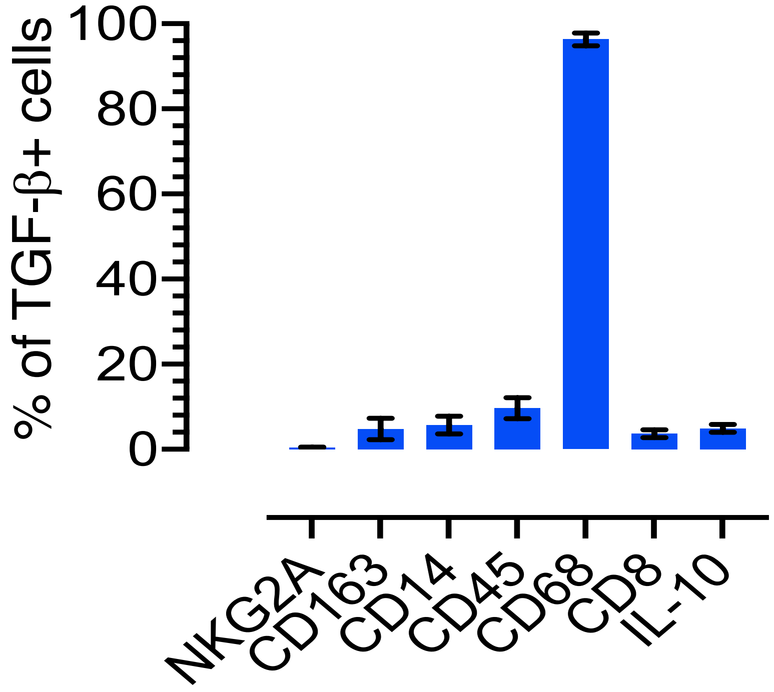

Supplemental Figure S3

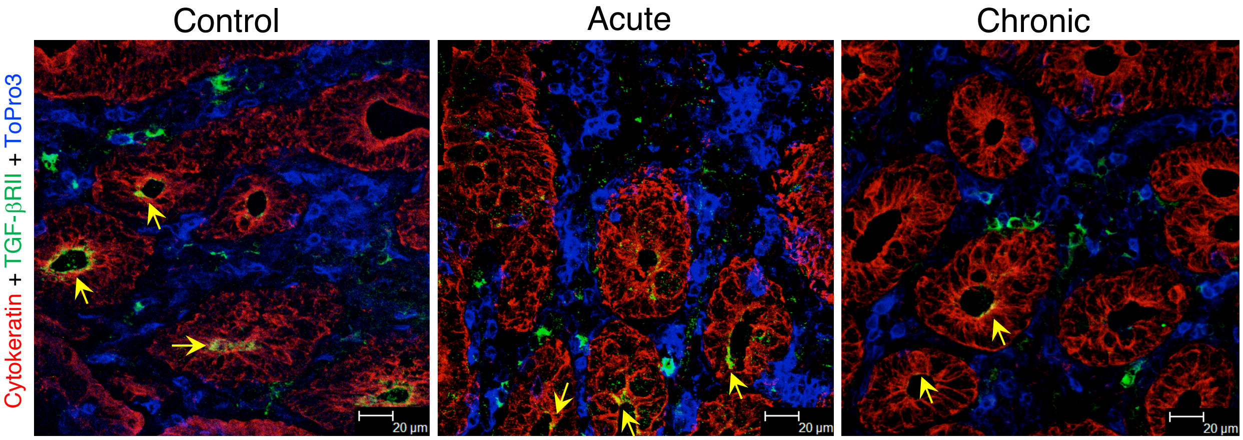

Supplemental Figure S4

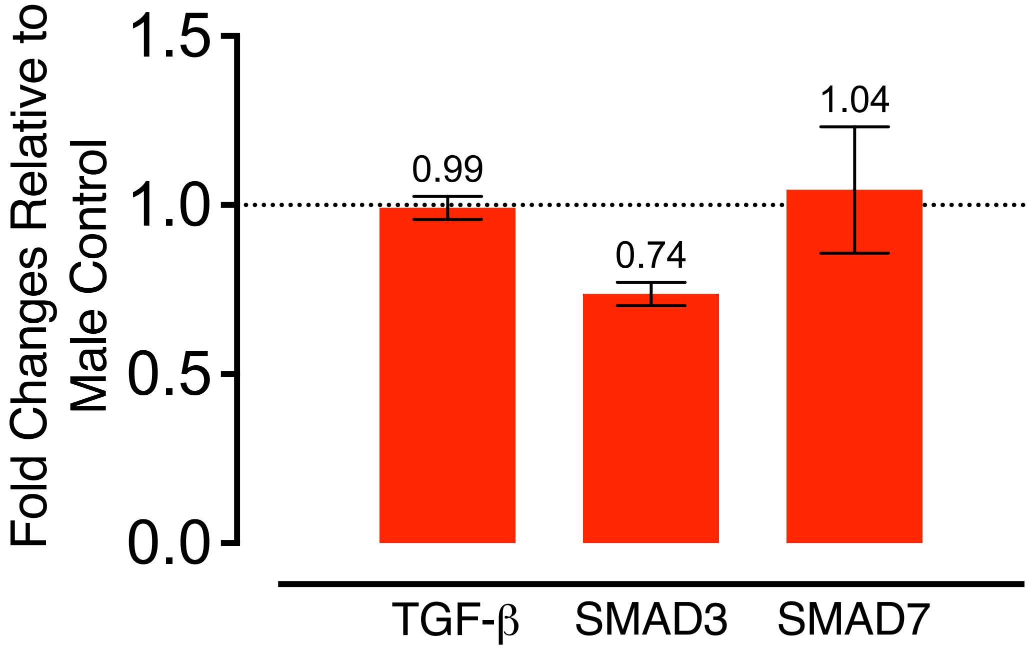

Supplement: Supplementary file 1 [file cells-10-00806-s001.zip › Supplemental Figures and Table/Supplemental FiguresS1-4.pdf]
